# Exposure to light enhances pre-adult fitness in two dark-dwelling sympatric species of ants

**DOI:** 10.1186/1471-213X-8-113

**Published:** 2008-12-02

**Authors:** Shahnaz Rahman Lone, Vijay Kumar Sharma

**Affiliations:** 1 Chronobiology Laboratory, Evolutionary and Organismal Biology Unit, Jawaharlal Nehru Centre for Advanced Scientific Research, PO. Box 6436, Jakkur, Bangalore 560 064, Karnataka, India

## Abstract

**Background:**

In insects, circadian clocks play a key role in enhancing fitness by regulating life history traits such as developmental time and adult lifespan. These clocks use environmental light/dark (LD) cycles to fine-tune a wide range of behavioral and physiological processes. To study the effect of environmental LD conditions on pre-adult fitness components, we used two dark-dwelling sympatric species of ants (the night active *Camponotus compressus *and the day active *Camponotus paria*), which normally develop underground and have fairly long pre-adult developmental time.

**Results:**

Our results suggest that ants develop fastest as pre-adults when maintained under constant light (LL), followed closely by 12:12 hr light/dark (LD), and then constant darkness (DD). While light exposure alters developmental rates of almost all stages of development, the overall pre-adult development in LL is speeded-up (relative to DD) by ~37% (34 days) in *C. compressus *and by ~35% (31 days) in *C. paria*. In LD too, development is faster (relative to DD) by ~29% (26 days) in *C. compressus *and by ~28% (25 days) in *C. paria*. Pre-adult viability of both species is also higher under LL and LD compared to DD. While pre-adult development time and viability is enhanced in LL and LD, clutch-size undergoes reduction, at least in *C. compressus*.

**Conclusion:**

Exposure to light enhances pre-adult fitness in two dark-dwelling species of *Camponotus *by speeding-up development and by enhancing viability. This suggests that social ants use environmental light/dark cycles to modulate key life history traits such as pre-adult development time and viability.

## Background

Circadian clocks maximize performance of a wide range of organisms by scheduling rhythmic behaviours at appropriate time of the day. These clocks help in anticipating rhythmic changes in the environment [1-3], in preparing for events such as migration and reproduction, and in maintaining perfect harmony between behavioural and metabolic cycles [4]. Mismatch between external and internal timings proves to be detrimental to the organism [5-7]. Malfunctioning circadian clocks have also been shown to have deleterious effect on respiration, behavior, and rate of aging in nematodes [8], and have been shown to cause severe mental disorders and physical discomfort in humans [9].

While we know a great deal about the structure and function of circadian clocks in a fairly wide range of organisms [10], their role in the regulation of life history traits such as pre-adult development time and adult lifespan still remains a mystery. It is generally believed that faster clocks speed-up development and shorten lifespan, and slower clocks slow-down development and lengthen lifespan [8,11-13]. In the fruit fly *Drosophila melanogaster *[11,13], melon fly *Bactrocera cucurbitae *[12], and nematode *Caenorhabditis elegans *[2], circadian clocks have been shown to regulate pre-adult development time.

Environmental light/dark regimes have been shown to have measurable effect on pre-adult development time in *Drosophila*; development is fastest in LL, followed by 12:12 hr LD, and then DD [11,14,15]. Most previous studies related to the effect of light regimes on developmental duration have been carried out in insects with relatively short development time, where light-mediated effects are not large enough to draw any meaningful conclusion.

Apart from pre-adult development time, egg-viability is also altered by environmental LD conditions [16]. For example, in the intestinal fluke of waterfowls *Echinostoma caproni*, it has been observed that eggs kept in darkness for up to 56 days do not hatch unless they are exposed to light [17], and frequency of egg-hatching increases with increasing light intensity [18]. In the maple aphid *Periphyllus californiensis*, eggs hatch much faster under shady sites than under sunny sites, because buds which the aphids feed upon open earlier in shady areas than sunny areas [19]. In the long-horned grasshopper *Metrioptera hime*, eggs hatch at higher rate under 12:12 hr LD compared to LL or DD [20]. This suggests that the extent and the rate of egg-hatching in insects depend upon environmental LD conditions, although the precise nature of such dependency may vary from species to species.

In *Camponotus *ants, pre-adult development time varies from species to species [21]; under natural conditions it ranges from one to three months, except in winters when pre-adult development is completely halted, particularly in boreal species (species living in Arctic and sub-Antarctic ecosystems). Some species of ants (*Formica polyctena*) also have the ability to raise their nest temperature using heat produced by their metabolic activities and by exposing their nests to direct sunlight [21]. This enables them to complete development within five to six weeks and get ready for their nuptial flights. The *Camponotus *ants on the other hand, are not known to use any exclusive thermoregulatory mechanism to regulate development [21]. This indicates that *Camponotus *ants use a different strategy to regulate their pre-adult development.

We assayed pre-adult fitness components such as development time and egg-viability in two dark-dwelling sympatric species of ants (the night active *C. compressus *and the day active *C. paria*) under three different light regimes (LL, 12:12 hr LD, and DD), to study the effect of light regimes on fitness, and to evaluate whether developmental response to environmental light/dark conditions is altered in these ants in the course of resource partitioning and sympatric speciation. The results provide interesting insights into light-dependent regulation of pre-adult fitness in ants.

## Results

We collected mated queens of two sympatric species of *Camponotus *ants (*C. compressus *and *C. paria*) while they were landing on the ground near their nuptial flight zones. These ants were introduced into three different light/dark regimes (LL, 12:12 hr LD, and DD) to study the effect of environmental light/dark conditions on pre-adult fitness components.

### 1. Effect of light/dark regimes on clutch-size and egg-viability

#### Clutch-size

**Figure 1:**
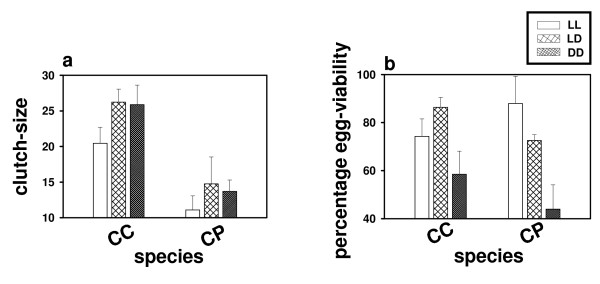
**Effect of light regimes on clutch-size and egg-viability**. a: In *Camponotus compressus *(CC), clutch-size is reduced under LL compared to LD and DD, whereas in *C. paria *(CP) it does not differ among three light regimes. Light regimes are plotted along the abscissa and clutch-size along the ordinate. Error bars represent 95% confidence interval around the mean for visual hypothesis testing. b: Percentage egg-viability of both species is greater under LL and LD compared to DD. Light regimes are plotted along the abscissa and percentage viability along the ordinate. Error bars represent 95% confidence interval around the mean for visual hypothesis testing.

Mated queens of each species were segregated into three groups and introduced into LL, LD, and DD, to estimate clutch-size of the first batch of eggs. While exposure to LL does not alter clutch-size in *C. paria*, it causes significant reduction in *C. compresses*. Two-way ANOVA on the clutch-size data showed a significant effect of light regime (*p *< 0.002) and species (*p *< 0.001); however, light regime × species interaction is statistically not significant (*p *= 0.52) (Table 1 and Fig. 1a). Post-hoc multiple comparisons using Tukey's HSD test revealed that in *C. compressus *clutch-size is significantly smaller in LL than in LD (*p *< 0.005) and DD (*p *< 0.005), while those under LD and DD do not differ (*p *> 0.05). In *C. paria*, effect of light regime on clutch-size is statistically not significant (*p *> 0.05); however, even in this species, a trend similar to that in *C. compressus *is observed.

**Table 1:** Results of two-way ANOVA on the clutch-size and egg-viability of *C. compressus *and *C. paria*.

	***df *Effect**	**MS Effect**	***df *Error**	**MS Error**	***F***	***p*-level**
*Clutch-size*						
						
Light regime (L)	2	128.31	67	17.73	7.23	< 0.002
Species (S)	1	1691.92	67	17.73	95.41	< 0.001
L × S	2	11.82	67	17.73	0.66	0.52
						
*Egg-viability*						
						
Light regime (L)	2	5781.66	67	200.23	28.87	< 0.001
Species (S)	1	320.81	67	200.23	1.61	0.21
L × S	2	1319.34	67	200.23	6.58	< 0.005

#### Egg-viability

Egg-viability is estimated as the number of eggs that successfully hatch as larvae. In both species, egg-viability is significantly enhanced in LL and LD compared to DD (Table 1 and Fig. 1b). ANOVA on the egg-viability data revealed a significant effect of light regime (*p *< 0.001) and light regime × species interaction (*p *< 0.005); however, effect of species is statistically not significant (*p *= 0.21) (Table 1 and Fig. 1b). Post-hoc multiple comparisons using Tukey's HSD test revealed that percentage viability of both species is significantly greater in LL (*p *< 0.001) and LD (*p *< 0.01) compared to DD, while those under LL and LD do not differ (*p *> 0.05).

### 2. Effect of light/dark regimes on the duration of pre-adult developmental stages

**Figure 2:**
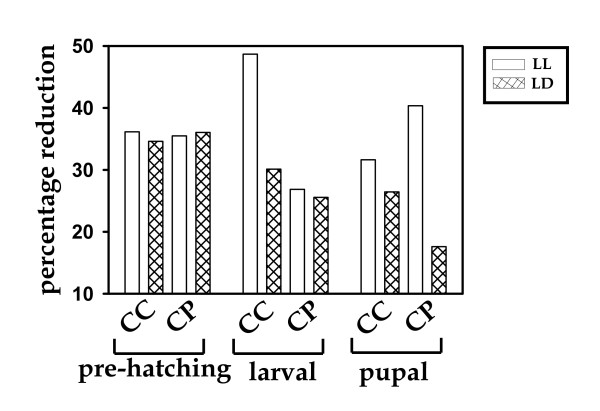
**Effect of light regimes on the pre-hatching, larval and pupal durations**. In LL, duration of pre-hatching stages is reduced (relative to DD) by ~36% in both *C. compressus *and *C. paria*, larval stages are reduced by ~49% in *C. compressus *and by ~27% in *C. paria*, and pupal stage is reduced by ~32% in *C. compressus *and by ~41% in *C. paria*. In LD, duration of pre-hatching stages is reduced (relative to DD) by ~36% in both *C. compressus *and *C. paria*, larval stages are reduced by ~30% in *C. compressus *and by ~26% in *C. paria*, and pupal stage is reduced by ~27% in *C. compressus *and by ~18% in *C. paria*. Species types are plotted along the abscissa and percentage reduction of the durations of specific developmental stages along the ordinate.

For each developmental stage (pre-hatching (L0), 1^st ^instar larval (L1), 2^nd ^instar larval (L2), 3^rd ^instar larval (L3), and pupal (P) stages), time of initiation of the stage was taken into account to estimate development time. In both species, duration of most pre-adult stages are significantly altered by light, albeit by different magnitudes (Tables 2, 3 and Fig. 2). In general, development is faster under LL, followed by LD, and then DD, in that order.

**Table 2:** Details of timing of initiation and duration of different developmental stages in *C. compressus*.

**Regimes**	**Number of colonies**	**Stages**	**Initiation of the stage in days** **(mean ± SD)**	**Duration of the stage in days** **(mean ± SD)**
LL	11	L0	N/A	22.08 ± 0.25
	11	L1	22.08 ± 0.25	03.25 ± 0.91
	11	L2	25.33 ± 0.85	04.50 ± 0.69
	11	L3	29.83 ± 1.21	05.81 ± 1.84
	11	P	35.64 ± 2.53	22.16 ± 1.84
	11	M	57.81 ± 3.41	N/A
				
LD	20	L0	N/A	22.61 ± 0.68
	20	L1	22.61 ± 0.68	03.61 ± 0.45
	20	L2	26.23 ± 0.79	04.06 ± 1.34
	19	L3	30.29 ± 1.66	10.81 ± 1.75
	18	P	41.10 ± 2.36	23.84 ± 3.37
	18	M	64.94 ± 3.36	N/A
				
DD	23	L0	N/A	34.58 ± 4.39
	23	L1	34.58 ± 4.39	05.68 ± 0.49
	23	L2	40.27 ± 4.28	06.52 ± 1.47
	21	L3	46.79 ± 3.49	11.83 ± 2.08
	18	P	58.62 ± 3.96	32.91 ± 3.58
	18	M	91.53 ± 4.20	N/A

Details of the timing of initiation and the duration of different developmental stages in *C. compressus*, under three light regimes. The pre-adult stages are indicated as L0 (pre-hatching), L1 (1^st ^instar), L2 (2^nd ^instar), 3rd instar (3^rd ^instar), P (pupal stage) and M (emergence). N/A refers to data not available.

**Table 3:** Details of timing of initiation and duration of different developmental stages in *C. paria*.

**Regimes**	**Number of colonies**	**Stages**	**Initiation of stage in days** **(mean ± SD)**	**Duration of stage in days** **(mean ± SD)**
LL	13	L0	N/A	23.28 ± 0.72
	13	L1	23.28 ± 0.72	04.80 ± 1.41
	13	L2	28.09 ± 1.78	03.33 ± 1.06
	13	L3	31.42 ± 2.02	06.86 ± 2.17
	11	P	38.29 ± 3.05	19.81 ± 2.16
	11	M	58.11 ± 2.46	N/A
				
LD	8	L0	N/A	23.08 ± 0.66
	8	L1	23.08 ± 0.66	05.70 ± 0.56
	8	L2	28.79 ± 0.79	02.62 ± 0.43
	8	L3	31.41 ± 0.97	06.94 ± 1.41
	6	P	38.35 ± 1.24	26.92 ± 1.83
	6	M	65.27 ± 2.08	N/A
				
DD	19	L0	N/A	36.09 ± 3.81
	19	L1	36.09 ± 3.81	05.88 ± 0.41
	19	L2	41.97 ± 3.83	06.54 ± 1.47
	19	L3	48.52 ± 4.38	08.08 ± 1.23
	9	P	56.60 ± 4.25	33.22 ± 3.50
	9	M	89.82 ± 3.60	N/A

Details of the timing of initiation and the duration of different development stages in *C. paria*, under three different light regimes. Other details as in Table 2.

#### Time of initiation of the first instar larvae

In both species, development of L1 is faster in LL and LD compared to DD (Tables 2, 3, 4). ANOVA on the L1 developmental time data revealed a significant effect of light regime (*p *< 0.001), while effects of species (*p *= 0.13) and light regime × species interaction are statistically not significant (*p *= 0.81) (Table 4). Post-hoc multiple comparisons using Tukey's HSD test showed that in both species L1 larvae develop significantly faster under LL and LD compared to DD (*p *< 0.001), while those in LL and LD do not differ (*p *> 0.05).

**Table 4:** Results of ANOVA on the pre-adult developmental time data.

	***df Effect***	**MS Effect**	***df *Error**	**MS Error**	***F***	***p*-level**
*1st instar (L1)*						
Light regime (L)	2	1640.51	88	8.53	192.13	< 0.001
Species (S)	1	20.4	88	8.53	2.39	0.13
L × S	2	1.77	88	8.53	0.2	0.81
						
*2nd instar (L2)*						
Light regime (L)	2	2198.17	88	8.7	252.5	< 0.001
Species (S)	1	65.19	88	8.7	7.48	< 0.01
L × S	2	4.81	88	8.7	0.55	0.58
						
*3rd instar (L3)*						
Light regime (L)	2	2889.83	85	8.53	338.73	< 0.001
Species (S)	1	36.14	85	8.53	4.23	< 0.05
L × S	2	13.42	85	8.53	1.57	0.21
						
*Pupal stage*						
Light regime (L)	2	3029.72	68	8.37	361.61	< 0.001
Species (S)	1	3.62	68	8.37	0.43	0.51
L × S	2	92.48	68	8.37	11.03	< 0.001
						
*Emergence*						
Light regime (L)	2	6857.4	68	11.82	579.66	< 0.001
Species (S)	1	0.97	68	11.82	0.08	0.78
L × S	2	15.98	68	11.82	1.35	0.27

Results of two-way ANOVA on the duration of different stages of pre-adult development (1^st ^instar, 2^nd ^instar, 3^rd ^instar, pupal stage and emergence), estimated from the egg stage.

#### Time of initiation of the second instar larvae

The trend of L2 development time is similar to those of L1 (Tables 2, 3, 4). Results of ANOVA on the L2 development time data revealed a statistically significant effect of light regime (*p *< 0.001) and species (*p *< 0.01), while effect of light regime × species interaction is statistically not significant (*p *= 0.58) (Table 4). Post-hoc multiple comparisons showed that L2 larvae take on average lesser time to develop in LL and LD than in DD (*p *< 0.001), while under LL and LD their development times do not differ (*p *> 0.05).

#### Time of initiation of the third instar larvae

The trend of third instar larval development time is similar to those observed for L1 and L2 larvae (Tables 2, 3, 4). ANOVA revealed a statistically significant effect of light regime (*p *< 0.001) and species (*p *< 0.05); however, light regime × species interaction is statistically not significant (*p *= 0.21) (Table 4). Turkey's HSD test for post-hoc multiple comparisons revealed that development time of third instar larvae is significantly reduced under LL and LD compared to DD (*p *< 0.001), while those in LL and LD do not differ (*p *> 0.05).

#### Time of initiation of the pupal stage

Light regime has a similar effect on pupal development time as that observed for previous stages (Tables 2, 3, 4). ANOVA on the pupal developmental time revealed a statistically significant effect of light regime (*p *< 0.001) and light regime × species interaction (*p *< 0.001); however, effect of species is statistically not significant (*p *= 0.51) (Tables 2, 3, 4). Multiple comparisons using Tukey's HSD test revealed that in *C. compressus *pupal development is faster under LL, followed by LD, and then DD (*p *< 0.001). In *C. paria*, pupae develop much faster in LL and LD compared to DD (*p *< 0.001), while developmental rates under LL and LD do not differ (*p *> 0.05).

#### Time of adult emergence

The pre-adult (egg to adult emergence) development time of both species differed significantly under three light regimes; development is fastest in LL, followed by LD, and then DD (Tables 2, 3, 4). ANOVA on the pre-adult development time data revealed a statistically significant effect of light regime (*p *< 0.001), while effects of species (*p *= 0.78) and light regime × species interaction are statistically not significant (*p *= 0.27) (Table 4). Post-hoc multiple comparisons using Tukey's HSD test suggests that both species develop faster in LL compared to LD (*p *< 0.05) and DD (*p *< 0.001).

To gain further insight into light regime effect on pre-adult development time of specific developmental stages we reanalyzed the development time data by broadly categorizing pre-adult duration into three stages: pre-hatching, larval and pupal stages, taking duration of the stage in question into account as opposed to the total time since egg stage. This was done to avoid effects of previous stages on the duration of the stage in question. Under LL, duration of pre-hatching stage is reduced by ~36% in *C. compressus *and *C. paria*, larval stage is reduced by ~49% in *C*. *compressus *and ~27% in *C*. *paria*, pupal stage is reduced by ~32% in *C*. *compressus *and ~41% in *C*. *paria *(Tables 2, 3 and Fig. 2). Overall, there is ~37% reduction in *C*. *compressus *and ~35% in *C. paria*. In LD also, duration of pre-adult developmental stages are reduced in both species; however, here the reductions are much smaller than those seen in LL.

## Discussion

Our studies have shown that clutch-size of the first batch of eggs in *C. compressus *is significantly reduced under LL and LD compared to DD, while in *C. paria *it does not differ among the three light regimes. Although, LL reduces reproductive output in *C. compressus*, it increases egg-viability in both species, which suggests that a sizable proportion of eggs laid in DD are nonviable trophic eggs [21]. These results are consistent with the findings of a previous study in *Tribolium*, where females are found to lay fewer eggs in LL than in DD [22]. Egg-hatching is inhibited under LL in the halibut *Hippoglossus hippoglossus *also [23], while in the intestinal fluke of waterfowls *Echinostoma caproni *it is inhibited in DD [18]. Apart from the presence and absence of light even the length of photoperiods have been shown to alter clutch-size; in the diamond back moth *Plutella maculipennis*, females lay more eggs when raised under long photoperiod (LD 15:9 hr) than under short photoperiod (LD 9:15 hr) [16]. In the leek moth *Acrolepia assectella*, females raised under LD (9:15 hr), LL, DD, have, ~8.3, ~4.1, and ~2.9 eggs, respectively, in their ovaries on the second day of emergence [16]. This suggests that light regimes play a crucial role in the modulation of fitness in a range of insect species.

In the ant species *C. herculeanus*, both worker and reproductive castes are produced around the same time of the year (during late summer or early winter) [21]; and during peak winter their pre-adult development is suspended completely. In a separate study, we observed that *Camponutus *ants exhibit seasonal preference in development; night active *C. compressus *develops faster under short (winter) days, while day active *C. paria *develops faster under long (summer) days (*Shahnaz Rahman Lone, Vinodh Ilangovan, Madhuvika Murugan and Vijay Kumar Sharma, unpublished manuscript*). This suggests that pre-adult development in ants is regulated by seasonal timers.

Temporal regulation of development is of prime importance in insects, as it controls a large number of critical processes such as cell cycle, growth of tissues, emergence of body patterns, formation of organs, and several postembryonic processes [24]. Therefore, even a small change in development may lead to catastrophic developmental defect, or may even cause appearance of a new phenotypic variant, which may be picked up by selection [24]. The results of our study suggest that exposure to LL and LD regimes speeds-up pre-adult development in two sympatric species of *Camponotus *ants. We also observed that light exerts large effects on the duration of several pre-adult stages, which clearly suggests that light-sensitive processes play a key role in the regulation of pre-adult development in *Camponotus *ants. While these results are consistent with the findings of a few previous studies in *Drosophila *[11,14,15], it is not clear to what extent circadian timing systems are involved in such developmental regulations.

The rate of pre-adult development in insects is believed to be regulated by multivariate interactions between light/dark cycles, temperature dependent developmental clocks, and temperature-compensated, light and temperature entrainable circadian clocks [25,26]. It is believed that circadian clocks, and/or LD cycles create "developmental gates" which periodically assess developmental states of insects; if an individual has completed certain developmental state by that time it is allowed to enter the next stage, otherwise it is forced to wait until the next gate opens [26]. Since circadian rhythms are abolished under LL such "developmental gates" would be absent, and individuals would be allowed to enter subsequent stages without any further delay; therefore, pre-adult development should be fastest in LL [26]. On the other hand in LD and DD regimes, developmental rates would be primarily determined by the "developmental gates"; in DD, circadian clocks of *Camponotus *ants free-run with a mean period which is often greater than 24 hr, and in LD it is 24 hr as clocks of these ants entrain to 12:12 hr LD cycles [27,28]. Based on the above considerations, pre-adult development in *Camponotus *ants would be fastest in LL, followed by LD, and then DD. While it is likely that LD regimes, and/or circadian clocks regulate development time, a possible role of social factors can not be completely ignored. There is a growing body of evidence to suggest that social insects have well developed species- and season-specific developmental strategies to meet challenges arising due to changing environmental conditions and social needs [28-30]. Such strategies have been reported earlier in honeybees; where nurse bees have been shown to have the ability to accelerate development and become foragers, in the event that a shortage of foragers arises in the colony [30]. It is unlikely that the amount of light and/or temperature associated with illuminating dark-adapted ants would cause changes in developmental rates by such large magnitudes, because both species are found to develop faster under 12:12 hr LD cycles than under 14:10 hr or 14:14 hr LD cycles (data not shown); although the amount of light per cycle in 12:12 hr LD cycle is 2 hr shorter than the other two LD regimes.

In an early study, Minis and Pittendrigh [31] had shown that the pink bollworm moth *Pectinophra gossypiella *undergoes faster embryonic development in LL compared to DD. In a number of insects the developmental rate is controlled by photoperiod [16]. For example, developmental rates of two species of cutworm *Agrostis *are significantly different under long and short photoperiods; *A. occulata *develops faster under long photoperiod, whereas *A. triangulum *develops faster under short photoperiod [16]. The results of the present study suggest that exposure to LL and LD reduces the duration of pre-hatching, larval, and pupal stages, in two dark-dwelling sympatric species of ants. The reductions in larval stage under LL and LD are by ~49% and ~30% in *C. compressus *and by ~27% and ~26% in *C. paria*. Similarly, the reductions in pupal stage under LL and LD are by ~32% and ~27% in *C. compressus *and by ~41% and ~18% in *C. paria*. To the best of our knowledge, developmental effects of light regimes, by such large magnitudes, have never been reported earlier in any species of insect.

While exposures to LL and LD regimes is found to speed-up pre-adult development in both day as well as night active species in a similar manner; in night active species reductions are more severe during larval stage, while in day active species it is more severe during pupal stage. In a separate study, when we assayed pre-adult development time under three different photoperiods (10:14 hr LD, 12:12 hr LD, and 14:10 hr LD); we found that while both species develop faster under 12:12 hr LD cycles, among the asymmetric photoperiods, night active *C. compressus *develops faster under long nights (10:14 hr), while day active *C. paria *develops faster under long days (14:10 hr) (*Shahnaz Rahman Lone, Vinodh Ilangovan, Madhuvika Murugan and Vijay Kumar Sharma, unpublished manuscript*). This suggests that while the core physiological effect of light is well conserved in both species, in the course of sympatric speciation they have evolved different developmental strategies to deal with seasonal changes in their environments.

Developmental plasticity is mediated by signals that cause the expression or repression of several genetic switches [24]. The key components of such switches comprise stage-specific heterochronic genes such as *lin14*, which causes developmental delays in the nematode *C. elegans*. The expression of the gene *lin4*, which binds to the 3' untranslated region of *lin14*, has been shown to prevent developmental delays [24]. In addition, the oscillating gene *lin42 *[24], a homolog of *Drosophila period *gene, is shown to regulate expression of both *lin4 *and *lin14*, through yet unknown mechanisms. It is not known yet if light signals modulate expression of such stage-specific heterochronic genes. Alternatively, LD regime effects could be mediated via darkness; because exposure to darkness is known to slow-down pre-adult development by lowering ecdysteriod levels [32], and acute reduction in ecdysteriod level causes complete inhibition of pupation [33]. The steroid hormone ecdysone is also known to play a crucial role in the molting of larval stages; as it sets the timing of pre-adult stages by binding to nuclear hormone receptors, which in turn regulate the expression of genes that govern development [34]. Further, it is possible that genes involved in pathways, such as notch, insulin and ecdysteroid signaling, mediate light-dependent developmental regulation in ants [35].

Social insect colonies are faced with numerous challenges arising due to fluctuating seasons, changing colony sizes, modifications in age structure, changes in nutritional requirement associated with colony development, changes in food availability, and predation pressure [30,36-40]. To meet such challenges, social insects have evolved "division of labour" [28,41], and developmental plasticity [30]. It is therefore likely that light-mediated developmental plasticity could be an evolutionary consequence of such ecological and social selection pressures.

In temperate environments where day/night lengths vary drastically throughout the year, photoperiodic signals play a key role in enhancing the fitness of insects [16]. A large number of insect species are believed to use photoperiodic signals to regulate their pre-adult development, an ability that forms a fundamental aspect of insect evolution. This idea is further corroborated by the findings of our study; where we show that in two dark-dwelling sympatric species of ants, clutch-size, egg-viability, and pre-adult development time are significantly altered by environmental light conditions. While reproductive fitness of one of the species is reduced in LL, egg-viability and developmental rates of both species are enhanced considerably. Our study suggests that development plasticity regulated by environmental light/dark conditions may provide an important dimension to the aspects related to fitness and overall success of social ants in adapting to diverse spatio-temporal niches.

## Conclusion

The ability to time physiological and behavioural processes using circadian and seasonal timers, and environmental light/dark cycles may play a crucial role in enhancing the overall fitness of social ants. Our study suggests that light manipulations may be an effective strategy for offsite conservation in ant species, where light-dependent regulations play a key role in the regulation of life history traits. The findings of our study also raise the possibility of boosting the production and turnover of economically important insect species through simple manipulations in the lighting schedules of the environment in which they are reared.

## Methods

Ground-dwelling ants are genetically programmed to dig deep into the soil to construct safe nests, where their progeny complete development [21]. The carpenter ant *C. compressus *forms its nest in forests; usually inside decomposed plants, and have evolved mutualism with aphids and coccids [21]. *C. compressus *and its sympatric partner *C. paria *undertake mating flights in the months of April-May-June. After mating they dig deep into the soil to form nests. The colonies of *C. paria *are found more than one and half feet deep under the soil, while those of *C. compressus *are located further deeper (*SRL and VKS, personal observation*).

Mated queens were collected from the Jawaharlal Nehru Centre for Advanced Scientific Research Bangalore campus (12° 58' N, 77° 40' E), soon after their mating flights, while they were landing on the ground. These ants were immediately transferred into petri plates (diameter × height = 90 mm × 15 mm), and were introduced into LL, 12:12 hr LD and DD regimes. These ants were labeled as CCLL, CCLD, CCDD, CPLL, CPLD, and CPDD, where CC and CP denote species names, and suffix indicate assay light regimes. The *C. compressus *queens are black in colour, 1.65 ± 0.07 cm (mean ± SD) long (head to back) and 0.39 ± 0.01 cm wide, while *C. paria *queens have yellowish-green bands on their abdomen and are slightly smaller in size (length: 1.31 ± 0.02 cm and width: 0.36 ± 0.04 cm).

White fluorescent light of 500 ± 20 lux intensity was used in LL, and during the light phase of LD; dim red light (λ > 640 nm) was used under DD, and during the dark phase of LD. Humidity (~75%) and temperature was kept constant by keeping ants in temperature controlled incubators (25 ± 1°C). Food in the form Bhatkar diet [42], and dilute honey solution was provided *ad libitum*. Fresh food was provided on the same day for all colonies. Observations were made with utmost care to avoid any disturbance to the queens and to the developing larvae. The numbers of queens and putative colonies used in the first set of experiments were CCLL (11), CCLD (18), CCDD (15), CPLL (11), CPLD (8), and CPDD (10). Eggs were counted regularly using a magnifying glass and fine brush until the numbers in a given batch became constant. Percentage viability was calculated as the percentage of eggs that successfully hatched as larvae, while those eggs that disappeared were treated as nonviable (trophic) eggs.

**Figure 3:**
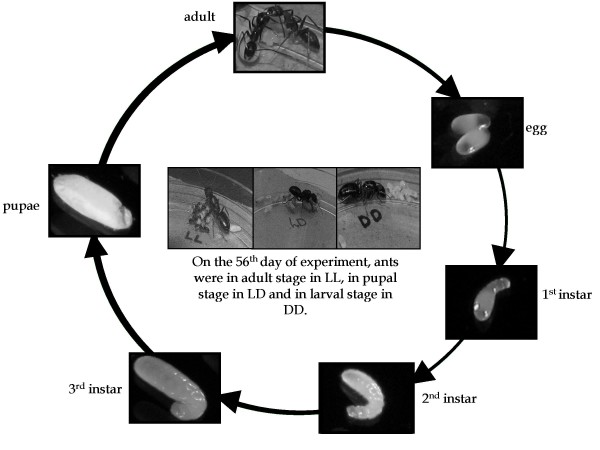
**Life cycle of *Camponotus *ants**. Shown in the centre are pictures of single queen colonies of *C. compressus *taken on the 56^th ^day of experiments (i.e. on the 56^th ^day after eggs were laid), for visual comparison of the magnitude of effects of light regimes on pre-adult development time. While adults can already be seen in the colony maintained under LL, those kept in LD and DD are still in pupal and larval stages, respectively. Shown along the circle are the pictures of adults, eggs, 1^st^, 2^nd^, 3^rd ^instar larvae, and pupae of *Camponotus compressus*.

The mean initiation time of 1^st^, 2^nd^, and 3^rd ^instar larvae, and of pupal and adult stages was used for the estimation of development time. First, second and third instar larvae of *C. compressus *are 1.05–1.35 mm (min-max), 1.40–2.00 mm, and 2.00–5.00 mm long, and 0.50–0.65 mm, 0.55–0.75 mm, and 0.85–1.40 mm wide; while those of *C. paria *are 1.00–1.30 mm, 1.35–2.00 mm, and 2.00–4.50 mm long, and 0.50–0.60 mm, 0.55–0.60 mm, 0.85–1.30 mm wide. The *C. compressus *pupae are 5.50–7.00 mm long and 2.00–3.00 mm wide; while those of *C. paria *are 5.00–5.20 mm long and 1.70–1.90 mm wide (Table 5). As seen in Figure 3, eggs, pupae, and freshly hatched larvae have distinct morphology, which made scoring developmental time of these stages easier. The timing of 2^nd ^and 3^rd ^larval instars was scored based on their sizes as described in Table 5. While there was hardly any overlap between the lengths of the three larval instars, in a few cases their breadth did overlap slightly. The 2^nd ^and 3^rd ^instar larvae were therefore considered to be in their subsequent stages, if on a particular day their measurements were greater than the maximum dimensions prescribed for the earlier stages, or else they were retained in their respective stages. The number of colonies used in these experiments is provided in Tables 2 and 3. Our study was carried out only on the first batch of eggs because it is this batch that is reared solely by the queen [21].

**Table 5:** Morphological measurements at different developmental stages of *Camponotus *ants.

		**Length (L) and Breadth (B) in millimeters**				
**Species**	**Parameter**	**Egg** **(min- max)**	**1st instar** **(min- max)**	**2nd instar** **(min- max)**	**3rd instar** **(min- max)**	**Pupal stage** **(min- max)**

*C. compressus*	Length (L)	1.00–1.20	1.05–1.35	1.40–2.00	2.00–5.00	05.5–7.00
	Breadth (B)	0.47–0.60	0.50–0.65	0.55–0.75	0.85–1.40	2.00–3.00
*C. paria*	Length (L)	0.90–1.20	1.00–1.30	1.35–2.00	2.00–4.50	5.00–5.20
	Breadth (B)	0.45–0.57	0.50–0.60	0.55–0.60	0.85–1.30	1.70–1.90

Morphological data of ants at different stages of their pre-adult development. Other details as in Table 2.

## Authors' contributions

SRL and VKS conceived the study, SRL performed the experiments, and both wrote the manuscript. Both the authors read and approved the final manuscript.
